# RETRACTED: Badr et al. Design and Evaluation of S-Protected Thiolated-Based Itopride Hydrochloride Polymeric Nanocrystals for Functional Dyspepsia: QbD-Driven Optimization, In Situ, In Vitro, and In Vivo Investigation. *Pharmaceuticals* 2023, *16*, 925

**DOI:** 10.3390/ph19040599

**Published:** 2026-04-08

**Authors:** Moutaz Y. Badr, Pratap Basim, Khaled M. Hosny, Waleed Y. Rizg, N. Raghavendra Naveen, Mallesh Kurakula, Fayez Alsulaimani, Awaji Y. Safhi, Fahad Y. Sabei, Mohammed Alissa, Abdulmohsin J. Alamoudi

**Affiliations:** 1 Department of Pharmaceutics, College of Pharmacy, Umm Al-Qura University, Makkah 24381, Saudi Arabia; mybadr@uqu.edu.sa; 2 ThermoFisher Scientific, Cincinnati, OH 45237, USA; pratapbasim@gmail.com; 3 Department of Pharmaceutics, Faculty of Pharmacy, King Abdulaziz University, Jeddah 21589, Saudi Arabia; wrizq@kau.edu.sa; 4 Center of Innovation in Personalized Medicine (CIPM), 3D Bioprinting Unit, King Abdulaziz University, Jeddah 21589, Saudi Arabia; 5 Department of Pharmaceutics, Sri Adichunchanagiri College of Pharmacy, Adichunchanagiri University, B.G Nagara, Karnataka 571448, India; raghavendra.naveen@gmail.com; 6 CURE Pharmaceutical, Oxnard, CA 93033, USA; 7 Department of Medical Laboratory Sciences, Faculty of Applied Medical Sciences, King Abdulaziz University, Jeddah 21589, Saudi Arabia; ffalsulaimani@kau.edu.sa; 8 Department of Pharmaceutics, College of Pharmacy, Jazan University, Jazan 45142, Saudi Arabiafsabei@jazanu.edu.sa (F.Y.S.); 9 Department of Medical Laboratory Sciences, College of Applied Medical Sciences, Prince Sattam bin Abdulaziz University, Al-Kharj 11942, Saudi Arabia; m.alissa@psau.edu.sa; 10 Department of Pharmacology and Toxicology, Faculty of Pharmacy, King Abdulaziz University, Jeddah 21589, Saudi Arabia; ajmalamoudi@kau.edu.sa

The journal retracts the article cited above, titled “Design and Evaluation of S-Protected Thiolated-Based Itopride Hydrochloride Polymeric Nanocrystals for Functional Dyspepsia: QbD-Driven Optimization, In Situ, In Vitro, and In Vivo Investigation” [1].

Following publication, concerns were brought to the attention of the Editorial Office regarding the integrity of a number of images presented in the publication.

Adhering to standard procedure, the Editorial Office and Editorial Board conducted an investigation that confirmed a duplication of Figure 3B in this publication [1] and Figure 3B in a previous publication [2], presenting different experimental conditions. While the authors collaborated in this process, raw material meeting the journal’s requirements for original images could not be provided for Editorial Board Evaluation. As a result, the Editorial Board has lost confidence in the reliability of the findings and has decided to retract this publication, as per MDPI’s retraction policy (https://www.mdpi.com/ethics#_bookmark30 (accessed on 28 December 2025)).

This retraction was approved by the Editor-in Chief of the journal *Pharmaceuticals*.

The authors do not agree to the retraction.
